# Mode-Division-Multiplexing of Multiple Bessel-Gaussian Beams Carrying Orbital-Angular-Momentum for Obstruction-Tolerant Free-Space Optical and Millimetre-Wave Communication Links

**DOI:** 10.1038/srep22082

**Published:** 2016-03-01

**Authors:** Nisar Ahmed, Zhe Zhao, Long Li, Hao Huang, Martin P. J. Lavery, Peicheng Liao, Yan Yan, Zhe Wang, Guodong Xie, Yongxiong Ren, Ahmed Almaiman, Asher J. Willner, Solyman Ashrafi, Andreas F. Molisch, Moshe Tur, Alan E. Willner

**Affiliations:** 1 Department of Electrical Engineering, University of Southern California, Los Angeles, CA 90089, USA; 2 School of Engineering, University of Glasgow, Glasgow G12 8QQ, Scotland, UK; 3 NxGen Partners, Dallas, Texas 75219, USA; 4 School of Electrical Engineering, Tel Aviv University, Tel Aviv 69978, Israel

## Abstract

We experimentally investigate the potential of using ‘self-healing’ Bessel-Gaussian beams carrying orbital-angular-momentum to overcome limitations in obstructed free-space optical and 28-GHz millimetre-wave communication links. We multiplex and transmit two beams (*l* = +1 and +3) over 1.4 metres in both the optical and millimetre-wave domains. Each optical beam carried 50-Gbaud quadrature-phase-shift-keyed data, and each millimetre-wave beam carried 1-Gbaud 16-quadrature-amplitude-modulated data. In both types of links, opaque disks of different sizes are used to obstruct the beams at different transverse positions. We observe self-healing after the obstructions, and assess crosstalk and power penalty when data is transmitted. Moreover, we show that Bessel-Gaussian orbital-angular-momentum beams are more tolerant to obstructions than non-Bessel orbital-angular-momentum beams. For example, when obstructions that are 1 and 0.44 the size of the *l* = +1 beam, are placed at beam centre, optical and millimetre-wave Bessel-Gaussian beams show ~6 dB and ~8 dB reduction in crosstalk, respectively.

Line-of-sight (LoS) wireless communication links are important for many applications, including backhaul, access, military and data centre environments^1^,^2^,^3^, and can exist through air or vacuum. Although today’s systems predominantly use radio frequency (RF) or millimetre waves, future systems may also employ a growing number of optical carrier waves to achieve higher capacities^4^,^5^,^6^,^7^. As in all communication systems, there is intense interest in techniques to increase capacities in both optical and millimetre-type systems.

One recently explored approach to significantly increase the capacity of free-space links is to spatially multiplex multiple orthogonal modes using a single transmitter/receiver aperture pair, in which each mode carries an independent data stream. Orthogonality ensures that the modes can be efficiently multiplexed at the transmitter, spatially co-propagated, and are demultiplexed at the receiver with minimal modal crosstalk.

Recent optical and millimetre-wave (mm-wave) demonstrations have shown that the structured beams carrying orbital-angular-momentum (OAM) can serve as a modal basis set for such a capacity-enhancing, mode-division-multiplexed (MDM), free-space system^8^,^9^. In 1992, it was discovered that OAM can be carried by an electromagnetic (EM) beam if its phase front ‘twists’ in a helical fashion^10^. The amount of OAM is defined by the (integer) number of 2π phase shifts that occur in the azimuthal direction, and beams with different OAM numbers form an orthogonal modal set^11^. OAM beams have a central intensity null and can be referred to as ‘vortex’ beams^12^. Multiple OAM beams can be multiplexed together, and OAM is compatible with multiplexing in other domains, such as polarization and wavelength multiplexing^13^.

A critical issue with all free-space LoS data links is the ability to overcome obstructions in the beam path^7^. Such a problem is exacerbated significantly in an OAM-multiplexed system, since obstructions will produce phase distortions in each beam’s phase front, thereby reducing the orthogonality and causing some amount of energy contained in a particular modal state to be coupled into other neighbouring modes^14^. Such distortion and crosstalk fundamentally affects OAM-multiplexed systems since the mode determines each unique channel, and phase perturbations can destroy the ability to recover each transmitted data channel uniquely. However, a key advance in multiple-channel, free-space links would be if the basic phase and amplitude profiles of orthogonal beams remain intact even if there is an obstruction in their path.

One potential approach could be to use Bessel-Gaussian (BG) beams for transmission over short ranges^15^,^16^,^17^,^18^,^19^,^20^,^21^,^22^,^23^. BG beams are propagation invariant over a length determined by the generation method and can extend to a few tens of metres^24^,^25^,^26^. BG beams have the unique property to reconstruct or ‘self-heal’ the transverse intensity and phase profiles after experiencing an obstruction. The self-healing property of the BG beams may have important applications in short-range, free-space communication links.

In this paper, we explore use of mode multiplexed BG beams in a free-space communication system that is fundamentally more tolerant to path obstructions. We demonstrate an MDM system of multiple BG beams carrying OAM for obstruction-tolerant optical and mm-wave high-speed links. In both the optical and the mm-wave links, two BG beams (*l* = +1 and +3), are multiplexed and transmitted over a distance of 1.4 m. In the optical regime, each beam carried 50-Gbaud quadrature-phase-shift-keying (QPSK) data, while each mm-wave beam carried 1-Gbaud 16-quadrature-amplitude-modulation (16-QAM) data. To emulate obstructed beam paths, opaque circular obstructions are used at various transverse positions. The ‘self-healing’ of BG beams is observed such that BG beams are able to reconstruct in the receiver planes. In both the optical and the mm-wave transmissions, we recover data channels and assess system performance in terms of crosstalk from neighbouring modes. As a comparison, the tolerance of non-Bessel OAM beams is investigated, under similarly obstructed path conditions. It is observed that under certain conditions, BG beams are more tolerant to obstructions than non-Bessel OAM beams. As an example, when obstructions are placed at beam centre, optical BG beams with *l* = +1 and *l* = +3 show >6 dB and >1.5 dB reduction in crosstalk as compared to optical non-Bessel OAM beams, respectively. When obstructions are placed off-centre, under similar conditions, optical BG beams with *l* = +1 and *l* = +3 show >3 dB and >8 dB reduction in crosstalk in comparison with optical LG beams, respectively. Similarly, mm-wave BG beams show >8 dB reduction in crosstalk as compared to the links employing non-Bessel OAM beams.

## Results

### Optical BG Beams

**Figure 1:**
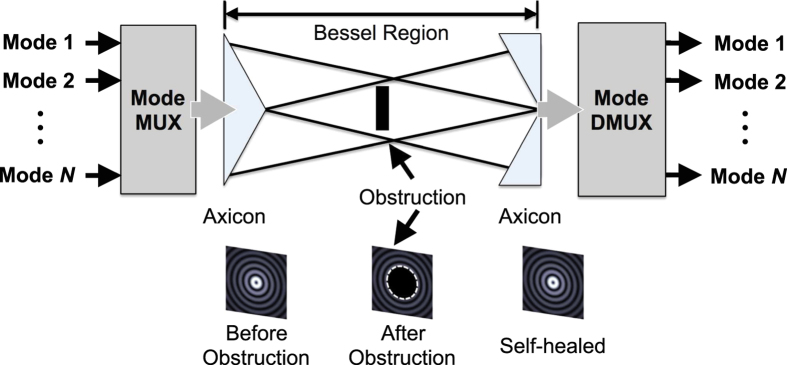
Conceptual block diagram of a multimodal link using multiplexed BG beams. ‘Bessel-region’ is the distance over which BG beams are propagation invariant and retain their profile. Insets depict transverse intensity profiles of a BG beam before and after an opaque disk and in the receiver plane.

Figure 1 shows a conceptual block diagram of a free-space link that uses spatially multiplexed data-carrying beams. *N* distinct input OAM beams, each carrying a distinct data channel, are spatially multiplexed and transmitted through an axicon to be transformed into BG beams. Within the ‘Bessel-region’, beams are propagation invariant and, therefore, can sustain partial obstructions. At the end of the Bessel-region, an exit axicon having opposite cone angles is placed to remove conical phases. Finally, an OAM mode demultiplexer separates each OAM beam. The ability of BG beams to sustain the adverse effects of an object inadvertently blocking the beam path within the Bessel region might be beneficial in recovering data channels.

**Figure 2:**
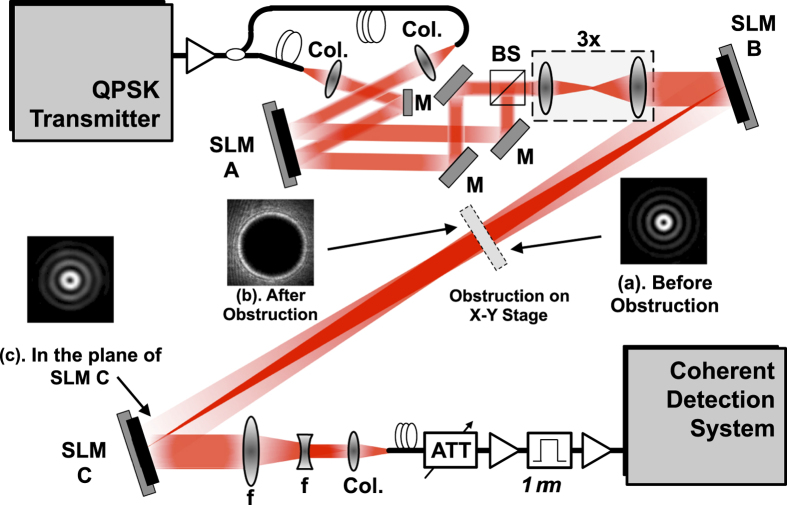
Optical Experimental Setup. Phase mask on SLM-B transforms multiplexed LG beams into BG beams. Opaque circular obstruction placed on the x-y stage partially obstructs multiplexed BG beams. Insets (**a**–**c**) show measured transverse intensity profiles of BG beam (*l* = 1) at different locations along the propagation direction, before and after an opaque circular obstruction of radius 1.5 mm. Col.: Collimator, f: Lens, M: Mirror, SLM: Spatial light modulator, BS: Beamsplitter, ATT: Attenuator.

**Figure 3:**
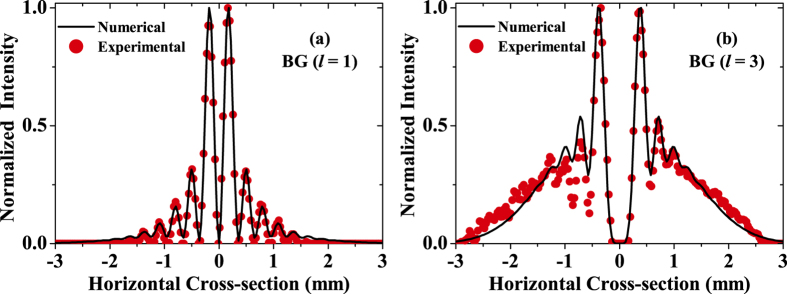
Measured intensity cross-sections of BG beams in the obstruction plane. BG beam with **(a)** *l* = 1, and **(b)** *l* = 3. Solid curves show numerically calculated cross-sections.

Figure 2 shows the experimental setup to demonstrate an obstruction-tolerant optical transmission. Two fundamental Gaussian beams, each carrying uncorrelated 50-Gbaud QPSK channels, are shone on two different regions of spatial light modulator ‘A’ (SLM-A) to be transformed into Laguerre-Gaussian (LG) beams with *l* = +1 and *l* = +3. The two LG beams are then multiplexed using a beamsplitter and passed through a 3× magnifying setup such that the LG beams with *l* = +1 and +3, in the plane of SLM-B, have spot sizes of 3.8 and 4.1 mm, respectively. The spot size of the LG beams is determined by measurements and subsequently calculating the second moment of the intensity using the expression^27^ 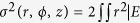

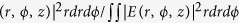
 in which, 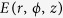 is the electric field of the beam expressed in polar coordinates at a plane located at distance z. Since LG beams can be transformed into BG beams by placing an axicon in the beam path^24^, SLM-B is used for this purpose. SLM-B is programmed with a phase mask whose transmittance function can be given by 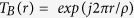 in which 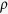 is an adjustable parameter controlling the cone angle of the holographic axicon^26^,^28^,^29^,^30^. After reflecting from SLM-B, the two LG beams transform into BG beams of similar mode orders (*l* = +1 and *l* = +3) and propagate to SLM-C. At a distance of 0.55 m from SLM-B, an opaque circular obstruction is placed on a linear translation stage such that it blocks the beam path at various transverse positions across the beam cross-section. Figure 3(a,b) show both experimentally measured and numerically calculated intensity cross-sections of the generated BG beams (*l* = +1, and *l* = +3) in the obstruction plane. The total distance between SLM-B and SLM-C is 1.4 m. To demultiplex one of the BG beams, SLM-C is programmed with a phase mask whose transmittance function is given by 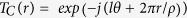. The phase mask on SLM-C effectively is a superposition of two phase masks. First, the 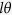 term removes the helical phases associated with OAM beams. The second term in the above transmission function represents a reversed axicon and is needed to remove the conical phases associated with the BG beams, such that the desired BG beam is converted back into a fundamental Gaussian beam for coupling into a single mode fibre (SMF)^31^. The received signal is then fed to a coherent receiver for detection and bit-error rate (BER) measurements.

**Figure 4:**
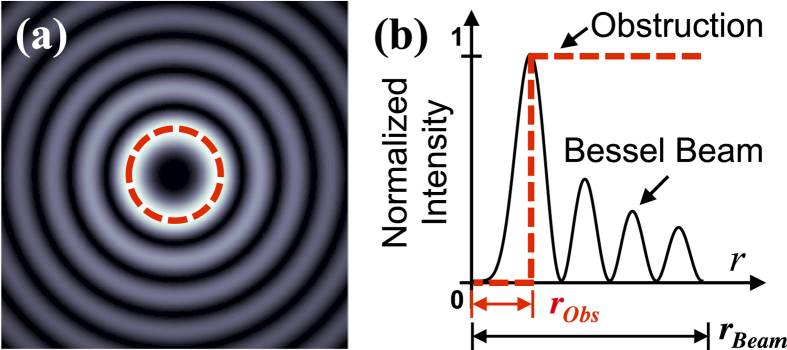
Pictorial depiction of an obstructed BG beam. (**a**) Transverse intensity profile of a BG beam. Dashed circle signifies opaque circular obstruction placed at the beam centre. In experiment, the obstruction is laterally moved to block the beam at various transverse positions. (**b**) Cross-section of BG beam intensity. Dashed line represents the cross-section of the opaque obstruction. Here 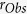 is the radius of the obstruction, and 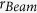 is the spot size of the beam as determined by measurements and subsequently calculating the second moment of the intensity.

As obstructions, the experiment uses opaque disks whose transmittance function is given by 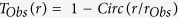
32, in which 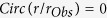 for 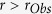, 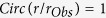 for 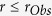, and 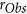 is the radius of the obstruction (see Fig. 4). In the obstruction plane, the measured spot sizes of BG beams *l* = +1 and +3 are 1 and 2.1 mm, respectively, which corresponds to the argument of the Bessel function 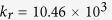
m^−1^. The spot sizes are measured using the relationship as given above. Instead of using only the size of the central lobe of the BG beams, we take into account the complete spot size of the BG beam including all the intensity rings. With this approach, the comparison with non-Bessel OAM beams is made based on the fraction of the obstructed beam by defining ζ as the obscuration ratio (i.e. ratio of obstruction radius to the radius of the beam, 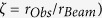. We chose two different opaque disks having radii 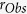 = 1 mm and 1.5 mm such that the obscuration ratios for *l* = +1 are 1 and 1.5, and for *l* = +3 are 0.47 and 0.71, respectively (see Table 1).

**Figure 5:**
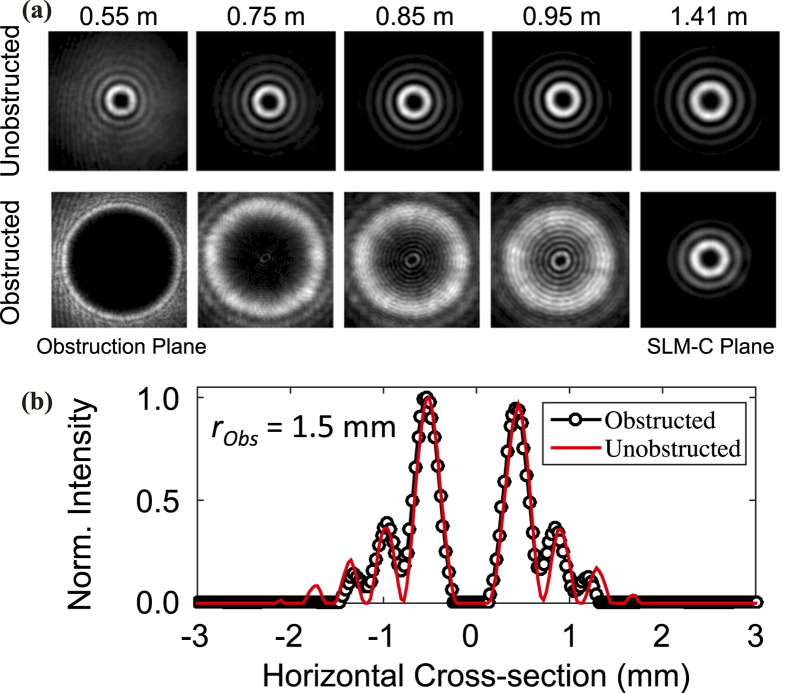
(**a**) Transverse intensity profiles of obstructed and unobstructed BG beam *l* = +3 after an obstruction of radius 1.5 mm (ζ = 0.71) at different locations along the propagation direction. **(b)** Normalized intensity cross–sections of obstructed and unobstructed beam in the plane of SLM–C.

While the link is operating, the obstructions are laterally traversed across the multiplexed beams. We observe that after encountering the obstructions, BG beams self-heal and reconstruct their transverse intensity profiles in the plane of SLM-C. As an example, Fig. 5(a) shows a comparison between the transverse intensity profiles of the obstructed and unobstructed BG beam with *l* = +3. In this figure, an obstruction of radius 1.5 mm (ζ = 0.71) is placed at the beam centre and images of the transverse intensity profiles are taken at various locations along the propagation direction. A comparison of obstructed and unobstructed beams in the plane of SLM-C (right-most column) reveals reconstruction of the BG beam. Furthermore, Fig. 5(b) shows the cross-sections of the normalized intensity profiles of both obstructed and unobstructed BG *l* = +3 in the plane of SLM-C. In this figure, we observe that the outer rings of the obstructed beam have diminished due to the power loss incurred by the obstruction. Nonetheless, the inner rings of the two beams are in good agreement.

To assess the link performance under obstructed path conditions, power in the desired mode and power leaked into the neighbouring modes (after coupling into the SMF) are measured by using appropriate phase masks on SLM-C, one at a time. For example, to measure power from *l* = +3 to *l* = +3 (P_33_, here P_*ij*_ refers to power received in the *j*-th mode while *i*-th mode is transmitted) and power leaked into *l* = +1 (P_31_), we transmit only one beam with *l* = +3 and place the obstruction in the beam centre. As the obstruction moves laterally across the beam, the power is measured in *l* = +3 (the desired mode). To measure the leakage power P_31_, SLM-C is loaded with the phase mask to select the undesired mode (*l* = +1). The same procedure is repeated for the case when *l* = +1 is transmitted, and received power is measured in the desired mode *l* = +1 (P_11_) and in the undesired mode *l* = +3 (P_13_).

**Figure 6:**
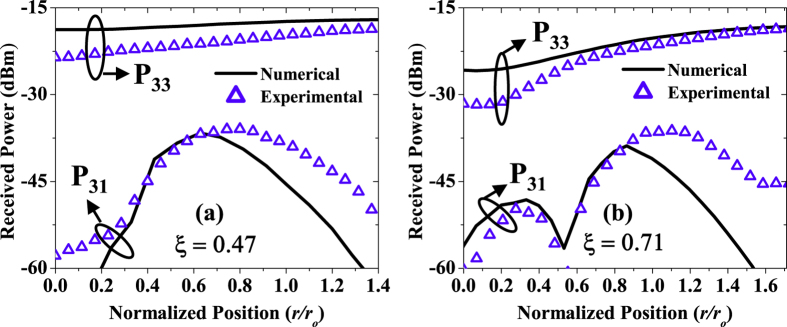
Received power as a function of normalized obstruction position for BG *l* = 3 obstructed by two obstructions of radii **(a)** 1 mm (ζ = 0.47), and **(b)** 1.5 mm (ζ = 0.71). Hollow triangles represent experimentally measured data and solid lines represent numerically calculated results. P_ij_ refers to the power received in j-th mode while i-th mode is transmitted.

Figure 6(a,b) shows the measured power as a function of normalized obstruction position. In these figures, the position 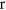 of the obstructions is normalized by the spot size of the beam, i.e.,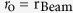. In the same figure, we also show the results obtained by the numerical simulations. There is a slight mismatch between the numerical and experimental data in the absolute sense, however, the overall trend matches. The slight mismatch is due to the ideal conditions considered in the model whereby the coupling to the SMF is neglected and the power received in each mode is calculated by the overlap integral between obstructed and unobstructed beams in the receiver plane.

To compare the performance of the obstructed BG beams with the obstructed LG beams (non-Bessel OAM beams), a second experiment is performed by slightly modifying the existing experimental setup. For the second experiment, the magnification setup is removed and SLM-B is replaced with a simple mirror. Additionally, in the absence of conical wavefront, a simpler phase mask is used on the SLM-C to convert LG beams back into fundamental Gaussian beams for coupling into SMF. The transfer function of the phase mask on the SLM-C can be given by 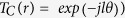. In this experiment, the spot size of the LG beams with *l* = +1 and *l* = +3 in the obstruction plane are 0.71 mm and 1 mm, respectively. To have obstructed path conditions similar to the BG case, the obstruction of radii 0.7 mm and 1 mm are chosen such that obscuration ratios for *l* = +1 are 1 and 1.4, and those for *l* = +3 are 0.7 and 1, respectively (see Table 1).

**Figure 7:**
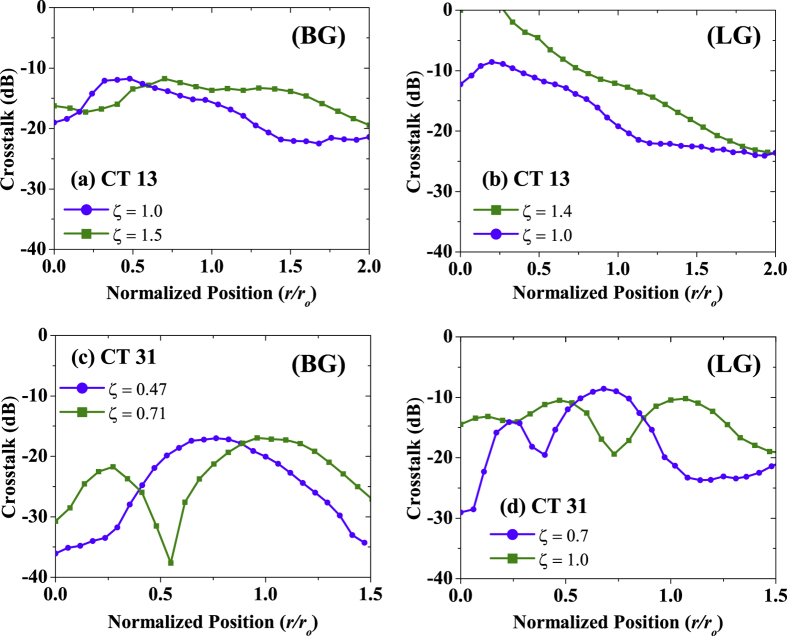
Crosstalk between different beams as a function of normalized obstruction position. CT ij refers to power leaked from i-th mode to the j-th mode. For comparison between BG and LG systems, obscuration ratios are also shown. The curves show crosstalk for: **(a)** BG *l* = +1 to *l* = +3, **(b)** LG *l* = +1 to *l* = +3, **(c)** BG *l* = +3 to *l* = +1, and **(d)** LG *l* = +3 to *l* = +1.

Figure 7 shows the measured crosstalk for the BG and LG cases. The crosstalk between the two modes is calculated by taking the ratio between the powers in the undesired and the desired mode. For the case in which BG beam with *l* = +1 is obstructed by an obstruction of radius 1 mm (ζ = 1) placed at the beam centre, we observe a crosstalk of −19 dB from *l* = +1 to *l* = +3 (Fig. 7(a)). The crosstalk in this case is 6.73 dB lower than the corresponding case for the LG beam as shown in Fig. 7(b). Higher crosstalk between LG modes can be explained by the inability of LG modes to reconstruct their beam profiles. As the obstruction size is increased to 1.5 mm (ζ = 1.5) for BG beam *l* = +1, a 2.7 dB increase in the crosstalk is seen. For the corresponding LG case, although obscuration ratio is smaller than the BG case (i.e. ζ = 1.4), system suffers from increased crosstalk. As the obstruction moves away from the beam centre, crosstalk decreases steadily. In terms of maximum value of crosstalk, BG system has 11.8 dB lower crosstalk than the LG system. Figure 7(c,d) shows the leakage power from beams with *l* = +3 to *l* = +1 when the two different obstructions are used to block BG and LG beams, respectively. Since the beams with *l* = +3 have larger spot size, obstructions of fixed radii result in smaller obscuration ratios. When the obstructions are placed in the beam centre, the BG system with ζ = 0.71 has a 1.6 dB lower crosstalk than the similar LG system. Similarly, the maximum crosstalk for the BG is ~17 dB, while for the LG system, the maximum crosstalk is ~9 dB.

**Figure 8:**
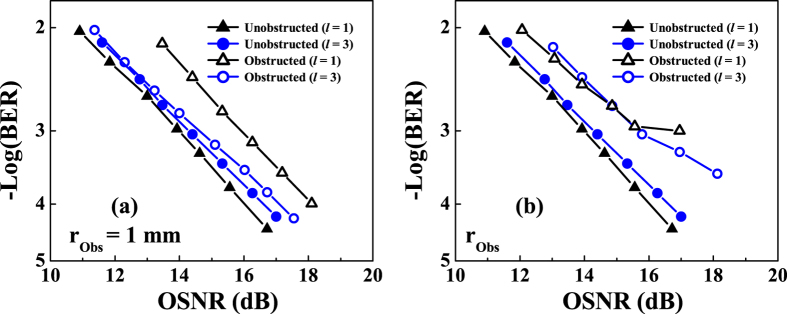
Measured BER for the multiplexed BG beams obstructed by obstructions of radii: (**a**) 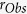 = 1 mm, and **(b)** 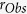 = 1.5 mm.

Figure 8(a,b) shows BER measurements for the unobstructed and the obstructed BG beams. First, the BER for both channels is measured without obstructing the beam path. Then, the BER is measured again, placing the obstruction in the beam centre, a location where maximum power loss occurs. As shown in Fig. 8(a,b), each channel can achieve a raw BER of 3.8 × 10^−3^. At a BER of 3.8 × 10^−3^, the optical signal-to-noise ratio (OSNR) penalties for the 1 mm and 1.5 mm obstructions are <2.3 dB and <1.7 dB, respectively. The large penalty for the channel on the *l* = +1 beam could be explained by noting that the *l* = +1 beam has a smaller spot size and, therefore, encounters a relatively larger obstruction as compared with the *l* = +3 beam. Note that for the LG system, the significant amount of crosstalk introduced by the obstructed beam path prevents the link from operating, and a BER measurement is therefore not possible.

### Millimetre-Wave BG Beams

**Figure 9:**
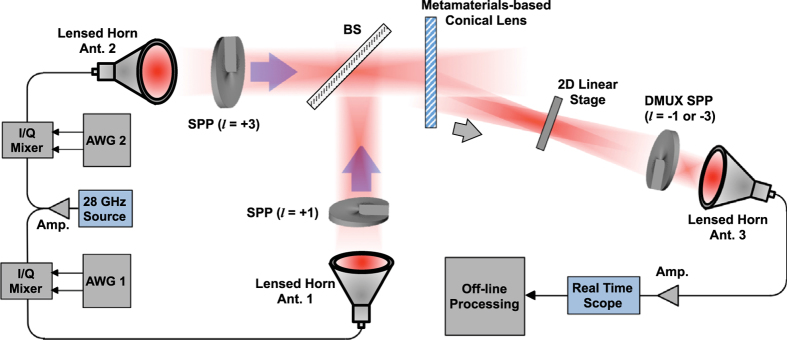
Millimetre-wave Experimental setup. 28-GHz Gaussian beams generated by lensed horn antennas are converted into OAM beams (*l* = 1 and 3) by using spiral phase plates and multiplexed by using a beamsplitter. Metamaterials-based axicon transforms multiplexed OAM beams into BG beams of similar mode orders. Received beams are demultiplexed one at a time by using an SPP having opposite spiral phase. Amp: amplifier, AWG: Arbitrary waveform generator, Ant.: Gaussian lensed horn antenna, BS: beamsplitter, SPP: spiral phase plate.

Figure 9 shows the experimental setup to demonstrate an obstruction-tolerant mm-wave link. A 28-GHz clock source is used to generate the carrier signal. Two arbitrary waveform generators (AWG1 and AWG2) are used to generate two 1-Gbaud 16-QAM signals. The two signals are then fed to horn antennas (Ant. 1 and Ant. 2), both of which are equipped with 15-cm convex lenses. A pair of spiral phase plates (SPPs) is used to transform the Gaussian beams into OAM beams with *l* = +1 and *l* = +3. The two OAM beams are multiplexed using a printed circuit board (PCB) based beamsplitter. To transform the *l* = +1 and *l* = +3 OAM beams into BG beams, a metamaterials-based axicon is used^33^. After passing through the axicon, the incident multiplexed OAM beams emerge as BG beams at 23.6 degrees angle, in the first diffraction order. Then, the multiplexed BG beams propagate toward the receiver antenna (Ant. 3) placed 1.4 m away from the metamaterials-based axicon. A 2D linear-translation stage holding mm-wave absorber of radius 6 cm is placed in the beam path at 0.4 m away from the axicon. At the receive end, an SPP having the opposite mode order is employed to demultiplex one of the multiplexed BG beams.

**Figure 10:**
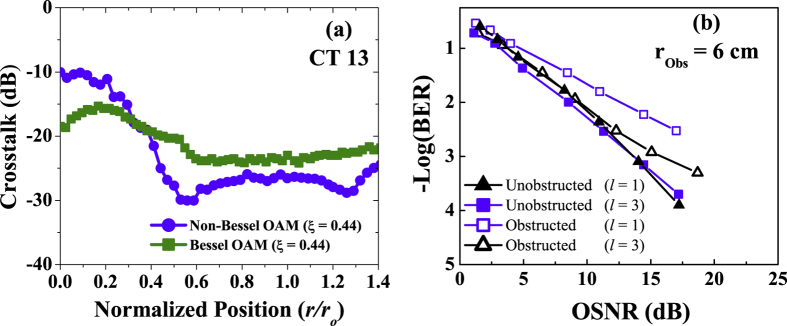
(**a**) Measured crosstalk from *l* = +1 to *l* = +3 for multiplexed millimetre-wave Bessel OAM and non-Bessel OAM beams. **(b)** Measured BER curves for the obstructed millimetre-wave Bessel OAM beams.

First, we characterize the power-transfer matrix between the OAM channels, following the same procedure as described in the optical section. Note that in the obstruction plane, the spot sizes of the BG *l* = +1 and *l* = +3 are 13.7 cm and 22.2 cm, respectively. To demonstrate obstructed non-Bessel OAM beams, the similar setup is used except for the axicon. The spot sizes of OAM beams in the obstruction plane are 18.1 cm and 26 cm, respectively. The obscuration ratios for the mm-wave BG and OAM beams are given in Table 2. Figure 10(a) shows the measured crosstalk of BG *l* = +1 as a function of the normalized obstruction position. We observe that when the obstruction is placed at beam centre, BG beams have 8.5 dB lower crosstalk than non-Bessel OAM beams. To investigate the influence of obstruction on the power penalty, Fig. 10(b) shows the BER curves for the two transmitted BG channels, both with and without obstruction. We observe power penalties <6 dB and <2 dB for the channels on BG *l* = +1 and *l* = +3 at a BER of 3.8 × 10^−3^, respectively.

## Discussion

When considering the future use of BG beams in free-space communication links, it would be beneficial to address some additional issues, including the following.

1. *Achievable link length*: In its simplest form, the length of the Bessel region for which there is self-healing is directly proportional to the spot size of the input beam and inversely related to the cone angle of the axicon^22^, meaning that a larger input beam spot size and a smaller axicon angle result in a longer Bessel region. Although many experiments have demonstrated the possibility of Bessel regions from a few metres to up to 100 m^25^,^26^, so-called ‘Bessel-like beams’ also exist with distance-dependant cone angles, allowing for long non-diffracting regions^34^.
2. *Relative location of obstruction along the link*: After experiencing an obstruction, the BG beam requires a certain distance to achieve self-healing such that the BG beam reconstructs its phase and amplitude profile. In the simple ray-optics limit, the minimum distance required by beams to self-heal after encountering an obstruction of diameter D can be given by D/2 tanθ, in which D is the obstruction diameter and 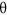 is related to the apex angle of the axicon^22^. In general, an obstruction may exist at any location along the beam path within the Bessel region, but there will likely exist scenarios with obstructions near the receiver for which full self-healing has yet to occur and that will produce a system penalty.
3. *Obstructions of different shapes:* In this work, we have assumed obstructions to be of circular symmetry; however, in reality, obstructions of any shape could exist. Based on the ray-optics principle, the self-healing of BG beams would occur for an obstruction of any shape as long as there remain *unobstructed* conical rays that interfere in some plane after the obstruction^20^,^22^,^35^. The effects of obstruction size and location across the beam cross-section should be further investigated and may produce effects on system performance that differ from our results.
4. *Alternate Methods:* While BG modes rely on their self-healing property to be sustained following the obstructions, other method may also be possible when the beams used are not propagation invariant. For such beams, one such method could be multiple-input multiple-output digital signal processing (MIMO-DSP) which estimates the received data based on the information contained in the neighbouring modes^36^,^37^. Effectiveness of this method has been demonstrated in optical links suffering from atmospheric turbulence; however, we believe that its performance in an obstructed path scenario has yet to be reported.
5. *Atmospheric Turbulence*: Atmospheric turbulence tends to distort both the intensity and phase profile of the OAM carrying beams^38^,^39^. Various methods have been proposed along with proof-of-concept lab experiments to compensate the effects of atmospheric turbulence on OAM-carrying beams^40^,^41^. Although we have not considered the effects of atmospheric turbulence on BG beams, however, schemes similar to those shown in the above referenced work could be considered to mitigate turbulence induced distortion and scintillation effects.

In addition, it is important to mention that other forms of propagation-invariant ‘Bessel-like’ beams also exist. For example, recently, it has been shown that LG beams with higher-order radial indices also possess self-healing properties^42^. Therefore, an investigation similar to the one reported in this paper could be performed with such other types of beams, evaluating relative performance gains.

## Methods

### Optical Experiment

A 50-Gbaud QPSK signal at 1550 nm wavelength is amplified using an EDFA and split into two paths. Two fibres of different lengths are employed to de-correlate the two signals. The two signals are coupled into free-space using collimators and are shone on an SLM with 600 × 792 pixels (pixel pitch of 20 micron), to convert Gaussian beams into LG beams. LG beams are multiplexed using beamsplitter and expanded by a magnifying setup. Another SLM with similar dimensions is used to convert multiplexed LG beams into multiplexed BG beams. At the receiver end, a third SLM demultiplexes beams one at a time. The demultiplexed beam is coupled into an SMF followed by an attenuator and an amplifier for noise loading. The signal is band pass filtered using a 1 nm optical filter, amplified and is fed to the coherent receiver for BER measurements.

### Millimetre-Wave Experiment

Two AWGs are employed to generate 1-Gbaud, 16-QAM baseband in-phase and quadrature (I and Q) signals. The baseband I/Q signals are up-converted to 28-GHz by mixing with a 28-GHz clock source. Two lensed horn antennas are used to launch mm-wave signal in free-space, which are then converted into OAM beams using a pair of SPPs. The two SPPs are fabricated using high-density polyethylene (HDPE), which has a refractive index n = 1.52 at 28-GHz. A PCB-based beamsplitter is used to combine the OAM beams. The multiplexed OAM beams are passed through a metamaterials-based axicon to be transformed into BG beams. At the receiver end, an SPP having opposite mode order is used to select one of the BG beams and couples demultiplexed beam into the receiver horn antenna. The received signals are recorded and digitized by an 80-Gsamples/s real-time scope, which has an analogue bandwidth of 32 GHz. An offline post-processing is carried out to calculate the BER for both channels.

## Additional Information

**How to cite this article**: Ahmed, N. *et al.* Mode-Division-Multiplexing of Multiple Bessel-Gaussian Beams Carrying Orbital-Angular-Momentum for Obstruction-Tolerant Free-Space Optical and Millimetre-Wave Communication Links. *Sci. Rep.* **6**, 22082; doi: 10.1038/srep22082 (2016).

## Supplementary Material

Supplementary Information

## Figures and Tables

**Table 1:** Obscuration Ratios for the Optical Link.

Beam Type	Mode Order	Spot Size	Obscuration Ratio (ζ)	
BG	*l* = 1	1 mm	1 (r_Obs_ = 1 mm)	1.5 (r_Obs_ = 1.5 mm)
	*l* = 3	2.1 mm	0.47 (r_Obs_ = 1 mm)	0.71 (r_Obs_ = 1.5 mm)
LG	*l* = 1	0.71 mm	1 (r_Obs_ = 0.7 mm)	1.4 (r_Obs_ = 1 mm)
	*l* = 3	1 mm	0.7 (r_Obs_ = 0.7 mm)	1 (r_Obs_ = 1 mm)

**Table 2:** Obscuration Ratios for the Millimetre-Wave Link.

Beam Type	Mode Order	Spot Size	Obscuration Ratio (ζ)
BG	*l* = 1	13.7 cm	0.44 (r_Obs_ = 6 cm)
	*l* = 3	22.2 cm	0.27 (r_Obs_ = 6 cm)
Non-Bessel OAM	*l* = 1	18.1 cm	0.44 (r_Obs_ = 8 cm)
	*l* = 3	26 cm	0.31 (r_Obs_ = 8 cm)

## References

[b1] Chan V. W. Free-space optical communications. IEEE J. Lightwave Technol. 24, 4750–4762 (2006).

[b2] Juarez J. C., Dwivedi A., Jones S. D., Weerackody V. & Nichols R. Free-space optical communications for next-generation military networks. IEEE Commun. Mag. 44, 46–51 (2006).

[b3] Hur S. *et al.* Millimeter wave beamforming for wireless backhaul and access in small cell networks. IEEE Trans. Commun. 61, 4391–4402 (2013).

[b4] Demers F., Yanikomeroglu H. & St-Hilaire M. A survey of opportunities for free space optics in next generation cellular networks. Paper presented at the *Ninth Annual Communication Networks and Services Research Conference (CNSR)*, Ottawa ON, Canada (doi: 10.1109/CNSR.2011.38) (2011, May 2–5).

[b5] Li Y. Pioro M. & Angelakisi V. Design of cellular backhaul topology using the FSO technology. Paper presented at the *2nd International Workshop on Optical Wireless Communications (IWOW),* Newcastle upon Tyne, UK (doi: 10.1109/IWOW.2013.6777766) (2013, June 6–10).

[b6] Navidpour S. M., Uysal M. & Kavehrad M. BER performance of free-space optical transmission with spatial diversity. IEEE Trans. Wireless Commun. 6, 2813–2819 (2007).

[b7] Kedar D. & Arnon S. Urban optical wireless communication networks: the main challenges and possible solutions. IEEE Commun. Mag. 42, S2–S7 (2004).

[b8] Wang J. *et al.* Terabit free-space data transmission employing OAM multiplexing. Nature Photon. 6, 488 (2012).

[b9] Yan Y. *et al.* High-capacity millimetre-wave communications with orbital angular momentum multiplexing. Nature Commun. 5, 4876 (2014). 25224763 10.1038/ncomms5876 PMC4175588

[b10] Allen L., Beijersbergen M. W., Spreeuw R. J. C. & Woerdman J. P. Orbital angular momentum of light and the transformation of Laguerre-Gaussian laser modes. Phys. Rev. A 45, 8185 (1992). 9906912 10.1103/physreva.45.8185

[b11] Yao A. M. & Padgett M. J. Orbital angular momentum: origins, behaviour and applications. Adv. Opt. Photon 3, 161 (2011).

[b12] Tamburini F. *et al.* Encoding many channels on the same frequency through radio vorticity: first experimental test. New J. Phys. 14, 033001 (2012).

[b13] Huang H. *et al.* 100 Tbit/s free-space data link enabled by three-dimensional multiplexing of orbital angular momentum, polarization, and wavelength. Opt. Lett. 39, 197–200 (2014). 24562105 10.1364/OL.39.000197

[b14] Franke-Arnold S. *et al.* Uncertainty principle for angular position and angular momentum. New J. Phys. 6, 103 (2004).

[b15] Gatto A., Tacca M., Martelli P., Boffi P. & Martinelli M. Free-space orbital angular momentum division multiplexing with Bessel beams. J. Opt. 13, 064018 (2011).

[b16] Ahmed N. *et al.* Experimental demonstration of obstruction-tolerant free-space transmission of two 50-Gbaud QPSK data channels using Bessel beams carrying orbital angular momentum. Paper presented at the *European Conference on Optical Communication (ECOC)*, Cannes, France (doi: 10.1109/ECOC.2014.6964157) (2014, Sept. 21–25).

[b17] Monk S., Arlt J., Robertson D. A., Courtial J. & Padgett M. J. The generation of Bessel beams at millimetre-wave frequencies by use of an axicon. Opt. Commun. 170, 213215 (1999).

[b18] Ahmed N. *et al.* Demonstration of an obstruction-tolerant millimeter-wave free-space communications link of two 1-Gbaud 16-QAM channels using Bessel beams containing orbital angular momentum. Paper presented at the *Third International Conference on Optical Angular Momentum (ICOAM),* New York, USA (2015, August 4–7).

[b19] Bouchal Z., Wagner J. & Chlup M. Self-reconstruction of a distorted nondiffracting beam. Opt. Commun. 151, 207 (1998).

[b20] Litvin I. A., McLaren M. G. & Forbes A. A conical wave approach to calculating Bessel–Gauss beam reconstruction after complex obstacles. Opt. Commun. 282, 1078 (2009).

[b21] Durnin J., Miceli J. J. Jr & Eberly J. H. Diffraction-free beams. Phys. Rev. Lett. 58, 1499 (1987). 10034453 10.1103/PhysRevLett.58.1499

[b22] McGloin D. & Dholakia K. Bessel beams: diffraction in a new light. Contemp. Phys. 46, 15 (2005).

[b23] Gori F., Guattari G. & Padovani C. Bessel-gauss beams. Opt. Commun. 64, 491 (1987).

[b24] Arlt J. & Dholakia K. Generation of high-order Bessel beams by use of an axicon. Opt. Commun. 177, 297 (2000).

[b25] Lin Y., Seka W., Eberly J. H., Huang H. & Brown D. L. Experimental investigation of Bessel beam characteristics. Appl. Opt. 31, 2708–2713 (1992). 20725197 10.1364/AO.31.002708

[b26] Paterson C. & Smith R. Higher-order Bessel waves produced by axicon-type computer-generated holograms. Opt. Commun. 124, 121 (1996).

[b27] Phillips R. L. & Andrews L. C. Spot size and divergence for Laguerre Gaussian beams of any order. Appl. Opt. 22, 643 (1983). 18195843 10.1364/ao.22.000643

[b28] Turunen J., Vasara A. & Friberg A. T. Holographic generation of diffraction-free beams. Appl. Opt. 27, 3959–3962 (1988). 20539499 10.1364/AO.27.003959

[b29] Vasara A., Turunen J. & Friberg A. T. Realization of general nondiffracting beams with computer-generated holograms. J. Opt. Soc. Am. A 6, 1748–1754 (1989). 2585173 10.1364/josaa.6.001748

[b30] Davis J. A., Carcole E. & Cottrell D. M. Intensity and phase measurements of nondiffracting beams generated with a magneto-optic spatial light modulator. Appl. Opt. 35, 593 (1996). 21069043 10.1364/AO.35.000593

[b31] Trichili A. *et al.* Detection of Bessel beams with digital axicons. Opt. Express 22, 17553–17560 (2014). 25090570 10.1364/OE.22.017553

[b32] Goodman J. W. Introduction to Fourier Optics. (Roberts and Company Publishers, 2005).

[b33] Zhao Z., Wang J., Li S. & Willner A. Metamaterials-based broadband generation of orbital angular momentum carrying vector beams. Opt. Lett. 38, 932–934 (2013). 23503264 10.1364/OL.38.000932

[b34] Belyi V., Forbes A., Kazak N., Khilo N. & Ropot P. Bessel–like beams with z–dependent cone angles. Opt. Express 18, 1966–1973 (2010). 20174026 10.1364/OE.18.001966

[b35] Milione G. *et al.* Measuring the self-healing of the spatially inhomogeneous states of polarization of vector Bessel beams. J. Optics 17, 035617 (2015).

[b36] Huang H. *et al.* Crosstalk mitigation in a free-space orbital angular momentum multiplexed communication link using 4 × 4 MIMO equalization. Opt. Lett. 39, 4360–4363 (2014). 25078177 10.1364/OL.39.004360

[b37] Ren Y. *et al.* Experimental demonstration of 16 Gbit/s millimeter-wave communications using MIMO processing of 2 OAM modes on each of two transmitter/receiver antenna apertures. Paper presented at the *Conference on Global Communications (GLOBECOM),* Austin TX, USA (doi: 10.1109/GLOCOM.2014.7037403) (2014, Dec. 8–12).

[b38] Paterson C. Atmospheric turbulence and orbital angular momentum of single photons for optical communication. Phys. Rev. Lett. 94, 153901 (2005). 15904145 10.1103/PhysRevLett.94.153901

[b39] Ren Y. *et al.* Atmospheric turbulence effects on the performance of a free space optical link employing orbital angular momentum multiplexing. Opt. Lett. 38, 4062–4065 (2013). 24321923 10.1364/OL.38.004062

[b40] Ren Y. *et al.* Adaptive-optics-based simultaneous pre-and post-turbulence compensation of multiple orbital-angular-momentum beams in a bidirectional free-space optical link. Optica 1, 376–382 (2014).

[b41] Xie G. *et al.* Phase correction for a distorted orbital angular momentum beam using a Zernike polynomials-based stochastic-parallel-gradient-descent algorithm. Opt. Lett. 40, 1197–1200 (2015). 25831291 10.1364/OL.40.001197

[b42] Mendoza-Hernández J., Arroyo-Carrasco M. L., Iturbe-Castillo M. D. & Chávez-Cerda S. Laguerre–Gauss beams versus Bessel beams showdown: peer comparison. Opt. Lett. 40, 3739–3742 (2015). 26274648 10.1364/OL.40.003739

