# Supplementary material for: Mode-Division-Multiplexing of Multiple Bessel-Gaussian Beams Carrying Orbital-Angular-Momentum for Obstruction-Tolerant Free-Space Optical and Millimetre-Wave Communication Links
Source: Sci Rep. 2016 Mar 1;6:22082. doi: 10.1038/srep22082 (PMC4772084; doi:10.1038/srep22082)
Supplement: Supplementary Information [file srep22082-s1.pdf]

Supplementary

Mode-Division-Multiplexing of Multiple Bessel-Gaussian Beams Carrying Orbital-Angular-Momentum for Obstruction-Tolerant Free-Space Optical and Millimetre-Wave Communication Links

Nisar Ahmed[1,*], Zhe Zhao[1], Long Li[1], Hao Huang[1], Martin P. J. Lavery[2], Peicheng Liao[1], Yan Yan[1], Zhe Wang[1], Guodong Xie[1], Yongxiong Ren[1], Ahmed Almaiman[1], Asher J. Willner[1], Andreas F. Molisch[1], Moshe Tur[3], and Alan E. Willner[1,*]

1. *Department of Electrical Engineering, University of Southern California, Los Angeles, CA 90089, USA.*
2. *School of Engineering, University of Glasgow, Glasgow G12 8QQ, Scotland, UK*
3. *School of Electrical Engineering, Tel Aviv University, Tel Aviv 69978, Israel*

Corresponding emails: nisarahm@usc.edu, willner@usc.edu

Section S.1 describes the numerical model to simulate the propagation of Bessel-Gaussian (BG) beams. The details of the metamaterials-based axicon used for the generation of millimetre-wave BG beams are presented in Section S.2.

S.1 Numerical Model for Optical Propagation

In this section, we present the numerical model used to calculate the results presented in Figures 3(a), 3(b), 6(a) and 6(b).

The electric field of a Laguerre-Gaussian (LG) beam at its waist is given by[1]:

$$E_{LG}(l,p,x_o,y_o,z_o)$$
$$= \frac{1}{w(z_o)}\sqrt{\frac{2p!}{\pi(|l|+p)!}}\left(\frac{\sqrt{x_o^2+y_o^2}\sqrt{2}}{w(z_o)}\right)^{|l|} e^{\frac{-x_o^2-y_o^2}{w(z_o)^2}} LP_p^{|l|}\left(\frac{2(x_o^2+y_o^2)}{w(z_o)^2}\right) e^{jl\left(tan^{-1}\left(\frac{y_o}{x_o}\right)\right)} \quad (1)$$

in which w is the beam waist, $LP_p^{|l|}$ are the generalized Laguerre polynomials, l and p are the azimuthal and radial mode indices, and x_o and y_o are the Cartesian coordinates in the waist plane at z_o. In order to transform an LG beam into a BG of same mode order, the LG beam is passed through an axicon. The electric field just after the axicon can be given by:

$$E_a(x_1, y_1, z_a) = E_{LG}(l, p, x_o, y_o, z_o)\, T_a\left(\sqrt{x_o^2 + y_o^2}\right) \tag{2}$$

where T_a is the transmission function of the axicon as given below[2]:

$$T_a\left(\sqrt{x_o^2 + y_o^2}\right) = exp(jk\gamma(n-1)\sqrt{x_o^2 + y_o^2}) \tag{3}$$

and $k = \frac{2\pi}{\lambda}$ is the wave number, n is the refractive index, and γ is the axicon opening angle.

The field given in (2) is numerically propagated to an obstruction plane located at a distance $z_{Obs} > z_a$ using Huygens-Fresnel diffraction integral. The numerical calculation is carried out by FFT-based implementation of the diffraction integral as described below[3]:

$$E_{Obs}(x_2, y_2, z_{Obs}) = \frac{j}{\lambda z_{Obs}} e^{\frac{-jk}{2z_{Obs}}(x_2^2+y_2^2)} FFT\left[E_a(x_1, y_1, z_a) e^{\frac{-jk}{2z_{Obs}}(x_1^2+y_1^2)}\right] \tag{4}$$

where z_{Obs} is the propagation distance from the axicon plane to the obstruction plane, FFT[.] is the Fast Fourier Transform operator, (x_1, y_1) and (x_2, y_2) are the Cartesian coordinates in the axicon and obstruction planes, respectively.

In order to model obstructed beam path, an opaque circular obstruction is introduced in the model such that the field just after the obstruction is given by:

$$E'_{Obs}(x_2, y_2, z_{Obs}) = E_{Obs}(x_2, y_2, z_{Obs})\, T_{Obs}\left(\sqrt{x_2^2 + y_2^2}\right) \tag{5}$$

The transmission function of the opaque obstruction can be given by[3]:

$$T_{Obs}\left(\sqrt{x_2^2 + y_2^2}\right) = 1 - Circ\left(\frac{\sqrt{x_2^2 + y_2^2}}{r_{Obs}}\right) \tag{6}$$

where $Circ\left(\frac{\sqrt{x_2^2+y_2^2}}{r_{Obs}}\right) = \begin{cases} 1\,; & \sqrt{x_2^2 + y_2^2} \geq r_{Obs} \\ 0\,; & \sqrt{x_2^2 + y_2^2} < r_{Obs} \end{cases}$.

After passing through the obstruction, the field is numerically propagated to the receiver plane using FFT method described in equation (4).

$$E_{Rx}(x_3, y_3, z_{Rx}) = \frac{j}{\lambda z_{Rx}} e^{\frac{-jk}{2z_{Rx}}(x_3^2+y_3^2)} FFT\left[E'_{Obs}(x_2, y_2, z_{Obs}) e^{\frac{-jk}{2z_{Obs}}(x_2^2+y_2^2)}\right] \tag{7}$$

We also calculate the unobstructed field at the receiver plane as given below:

$$E'_{Rx}(x_3, y_3, z_{Rx}) = \frac{j}{\lambda z_{Rx}} e^{\frac{-jk}{2z_{Rx}}(x_3^2+y_3^2)} FFT\left[E_{Obs}(x_2, y_2, z_{Obs}) e^{\frac{-jk}{2z_{Obs}}(x_2^2+y_2^2)}\right] \tag{8}$$

Finally, to calculate the received power in the desired mode and power coupled in the neighbouring modes, overlap integral is calculated using the expression given below:

$$P_{Rx} = \frac{\left|\iint E'^{*}_{Rx}\, E_{Rx}\, dx_3 dy_3\right|^2}{\iint |E'_{Rx}|^2 dx_3 dy_3 \quad \iint |E_{Rx}|^2 dx_3 dy_3} \tag{9}$$

S.2 Design of Metamaterials-based Axicon for 28 GHz Frequencies

This section presents the details of the millimetre-wave generation of BG beams using metamaterials-based axicon. Supplementary Figs. 1(a) and 1(b) show the geometric parameters and schematic structure of the designed metamaterials-based axicon. Using standard printed circuit board (PCB) processing, we etch spatially varying slit arrays in a copper film on a single-layer PCB. The period of each unit is 3.5 mm and the size of each sub-wavelength rectangular slit is 3.06×0.68 mm. The top layer of the designed PCB is covered by tin-lead-solder copper with thickness of 0.04 mm. The substrate is made of laminate with thickness of 1.5 mm and refractive index of 2.04 at 28-GHz. The subwavelength rectangular slit arrays can enhance the transmission of linearly polarized light perpendicular to the slit direction[4], (i.e., angle α relative to x direction). Each unit can be regarded as a localized linear polarizer and hence introduces a so-called Pancharatnam–Berry phase to the output beams[5]. The angle α can be tuned to control the phase of the output beams. Assuming a linearly polarized beam with Jones vector $E_{in} = [1 \quad 0]^T$ as the input beam, the output beam can be given by:

$$\boldsymbol{E}_{out} = 0.25\left\{e^{j2\alpha}\begin{bmatrix}1 & -j\end{bmatrix}^{T} + e^{-j2\alpha}\begin{bmatrix}1 & j\end{bmatrix}^{T}\right\} + 0.5\begin{bmatrix}1 & 0\end{bmatrix}^{T} \tag{10}$$

Equation 10 shows that the output beam consists of three polarization states, namely right circular polarization $[1 \quad -j]^T$, left circular polarization $[1 \quad j]^T$ and a linear polarization state $[1 \quad 0]^T$.

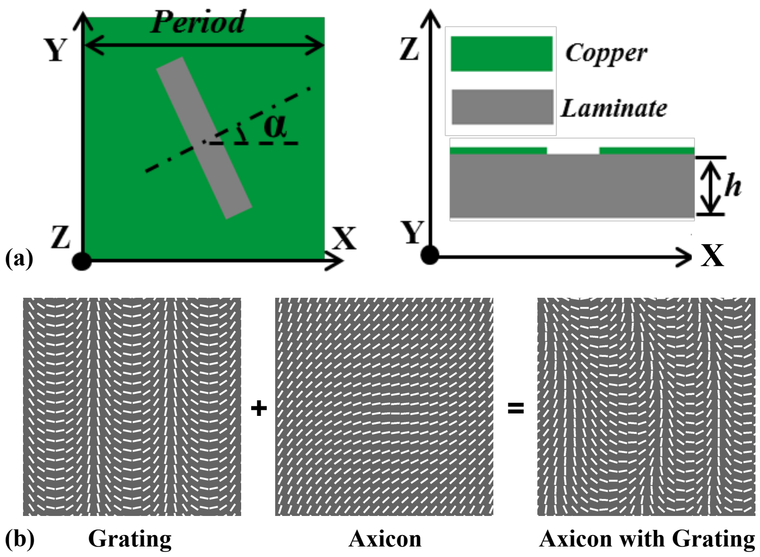

Figure 1. (a) Design parameters, and (b) schematic structure of the metamaterials-based axicon for BG beam generation. The orientation angle is α = πx/Λ + πηr, where Λ = 2.72 cm and η = 0.303 cm^{-1}. The size of the subwavelength rectangular slits is 3.06×0.68 mm.

We also notice that the output components of right and left circular polarization states have phases $e^{j2\alpha}$ and $e^{-j2\alpha}$ related to angel α, respectively, suggesting that rectangular slit arrays with spatially varying angle can be used as a desired phase mask[6]. Supplementary Fig. 1(b) shows a schematic structure of the metamaterials-based axicon to generate the BG beams. The axicon can be regarded as a phase mask whose phase shift decreases linearly with the radial distance r. The transmission function of the axicon can be written as[7]:

$$T = exp\left(-i\frac{\beta r}{\lambda}\right) \tag{11}$$

where λ is the wavelength of the incident beam. The parameter β determines the dependence of phase shift on the radial distance r. In order to use the rectangular slit arrays as an axicon, we set the angle α as a function of the radial distance r, namely, α = βr/2λ. Consequently, if $E_{in} = [1 \quad 0]^T$ is used as the input beam, the output beam becomes:

$$\boldsymbol{E}_{out} = 0.25\left\{e^{j\beta r/\lambda}\begin{bmatrix} 1 & -j \end{bmatrix}^{\mathrm{T}} + e^{-j\beta r/\lambda}\begin{bmatrix} 1 & j \end{bmatrix}^{\mathrm{T}}\right\} + 0.5\begin{bmatrix} 1 & 0 \end{bmatrix}^{\mathrm{T}} \tag{12}$$

Equation 12 shows that the designed axicon introduces a phase shift of $e^{(-j\beta r/2\lambda)}$ to the left circularly polarized components such that left circularly polarized output beam forms a BG beam. In order to separate the generated

Bessel beam from other components of the output beam, a grating structure is introduced in the x direction (see Supplementary Fig. 1(b)). As a result, the angle α can be expressed as $\alpha = \pi x/\Lambda + \pi\eta r$, in which, $\Lambda = 2.72$ cm and $\eta = 0.303$ cm^{-1}. By using this arrangement, a normally incident 28-GHz beam is transformed into a BG beam and emerges at 23.6 degrees relative to the incident direction.

References

1. Yao, A. M. and Padgett, M. J. Orbital angular momentum: origins, behaviour and applications. *Adv. Opt. Photon.* **3,** 161 (2011).
2. McGloin, D. et al. Three-dimensional arrays of optical bottle beams. *Opt. Commun.* **225,** 215-222 (2003).
3. Goodman, J. W. *Introduction to Fourier Optics*. (Roberts and Company Publishers, 2005).
4. Ruan, Z. and Qiu, M. Enhanced Transmission through Periodic Arrays of Subwavelength Holes: The Role of Localized Waveguide Resonances. *Phys. Rev. Lett*. **96,** 233901 (2006).
5. Yu, N. and Capasso, N. Flat optics with designer metasurfaces. *Nature Mater.* **13,** 139-150 (2014).
6. Zhao, Z., Wang, J., Li, S. and Willner, A. Metamaterials-based broadband generation of orbital angular momentum carrying vector beams. *Opt. Lett.* **38,** 932-934 (2013).
7. Pérez, M. V., Gómez-Reino, C. and Cuadrado, J. M. Diffraction patterns and zone plates produced by thin linear axicons. *Optica Acta* **33,** 1161–1176 (1986).
